# Profiles of resting state functional connectivity in temporal lobe epilepsy associated with post-laser interstitial thermal therapy seizure outcomes and semiologies

**DOI:** 10.3389/fnimg.2023.1201682

**Published:** 2023-11-09

**Authors:** Mashaal Syed, Jingya Miao, Anish Sathe, Kichang Kang, Arichena Manmatharayan, Michael Kogan, Caio M. Matias, Ashwini Sharan, Mahdi Alizadeh

**Affiliations:** ^1^Department of Neurological Surgery, Vickie and Jack Farber Institute for Neuroscience, Thomas Jefferson University, Philadelphia, PA, United States; ^2^Department of Neurology, Detroit Medical Center, University Health Center, Detroit, MI, United States; ^3^Department of Neurological Surgery, University of New Mexico, Albuquerque, NM, United States; ^4^Thomas Jefferson Integrated Magnetic Resonance Imaging Center, Department of Radiology, Thomas Jefferson University, Philadelphia, PA, United States

**Keywords:** temporal lobe epilepsy, laser interstitial thermal therapy, functional connectivity, seizure freedom, focal aware seizures, focal impaired awareness seizures

## Abstract

**Introduction:**

It is now understood that in focal epilepsy, impacted neural regions are not limited to the epileptogenic zone. As such, further investigation into the underlying functional connectivity (FC) patterns in those enduring Temporal Lobe Epilepsy (TLE) with Mesial Temporal Sclerosis (MTS) is imperative to understanding the intricacies of the disease.

**Methods:**

The rsfMRIs of 17 healthy participants, 10 left-sided TLE-MTS patients with a pre-operative history of focal impaired awareness seizures (FIA), and 13 left-sided TLE-MTS patients with a pre-operative history of focal aware seizures (FA) were compared to determine the existence of distinct FC patterns with respect to seizure types. Similarly, the rsfMRIs of the above-mentioned healthy participants, 16 left-sided TLE-MTS individuals who were seizure-free (SF) 12 months postoperatively, and 16 left-sided TLE-MTS persons without seizure freedom (nSF) were interrogated. The ROI-to-ROI connectivity analysis included a total of 175 regions of interest (ROIs) and accounted for both age and duration of epileptic activity. Significant correlations were determined via two-sample *t-*tests and Bonferroni correction (α = 0.05).

**Results:**

Comparisons of FA and FIA groups depicted significant correlations between the contralateral anterior cingulate gyrus, subgenual region, and the contralateral cerebellum, lobule III (*p*-value = 2.26e-4, mean z-score = −0.05 ± 0.28, T = −4.23). Comparisons of SF with nSF depicted two significantly paired-ROIs; the contralateral amygdala and the contralateral precuneus (*p-*value = 2.9e-5, mean z-score = −0.12 ± 0.19, T = 4.98), as well as the contralateral locus coeruleus and the ipsilateral intralaminar nucleus (*p*-value= 1.37e-4, mean z-score = 0.06 ± 0.17, T = −4.41).

**Significance:**

FC analysis proves to be a lucrative modality for exploring unique signatures with respect to seizure types and postoperative outcomes. By furthering our understanding of the differences between epileptic phenotypes, we can achieve improvement in future treatment modalities not limited to targeting advancements.

## Introduction

Temporal Lobe Epilepsy (TLE) is the most common type of focal seizure diagnosed in tertiary centers, as well as a form of medically-refractory epilepsy routinely referred for surgical intervention (Abarrategui et al., 2021). Mesial Temporal Sclerosis (MTS), also known as hippocampal sclerosis, is a frequent histopathological finding and a typical cause of TLE in adults (Asadi-Pooya et al., 2017; Blumcke et al., 2017). In just the United States alone, approximately 143,000–191,000 people who suffer from refractory-TLE with MTS can benefit from surgery (Asadi-Pooya et al., 2017). Although the standard surgical intervention for TLE is Anterior Temporal Lobectomy, other minimally invasive and non-resective options like Laser Interstitial Thermal Therapy (LiTT) have gained popularity in recent years. Despite the known efficacy of surgery, these interventions have each shown varying rates of favorable outcomes in patients; the basis for which is not yet well understood (Chang et al., 2015). It is suggested that heterogeneity in treatment and evaluation pre and post-surgery, as well as patient-specific clinical factors, may account for these differences (Mohan et al., 2018).

Functional connectivity (FC) through fMRI uses temporal synchronization of fluctuating blood-oxygen-level-dependent (BOLD) signals to correlate activity in different regions of the brain (Friston, 1994; Eickhoff and Müller, 2015). This technique has been used to detect physiologic connections in healthy individuals as well as pathologic alterations in a wide range of different disease states, such as Parkinson's Disease, spinal cord injury, and, notably, different types of epilepsy (Kaushal et al., 2017; Younce et al., 2021). In TLE patients, these patterns of connectivity have been paired with clinical data to reveal functional alterations underlying different seizure presentations. Of note, both FA and FIA seizure semiology are primarily differentiated based on clinical phenotypes; the cognitive impairment and loss of consciousness associated with the latter have been linked to diffuse brain network changes (Englot et al., 2010). Considering that alertness is a fundamental aspect of cognition, and the locus coeruleus (LC) is closely related to alertness, prior literature has also examined differences in locus coeruleus connectivity in patients with FA and FIA seizures (Liu et al., 2020). Overall, studying alterations in resting-state brain FC could be conducive to exploring the mechanisms underlying cognitive dysfunction in individuals with epilepsy.

Moreover, FC analysis on preclinical resting-state fMRI data has been used to predict clinical outcomes in epilepsy patients, indicating that pathological alterations in epilepsy also may differ between patients who responded to temporal lobe surgery by remaining seizure-free (SF) and those who did not (nSF) (DeSalvo et al., 2020). The success of focal epilepsy surgery relies on an accurate and complete delineation of the epileptogenic zone. It is identified by intracranial electroencephalogram during a seizure event, and surgical removal of the epileptogenic zone or disruptions of its networks is associated with improved clinical outcomes (Nissen et al., 2017; Ibrahim et al., 2018; D'Cruz et al., 2019; Neal et al., 2019). However, about a third of patients still fail to achieve postoperative seizure freedom, likely due to incomplete delineation and understanding of both regional and global brain networks (Reid and Staba, 2014; Englot et al., 2015; Nissen et al., 2017; Neal et al., 2019). Previous studies have demonstrated that nSF patients tend to present with larger widespread networks, increased connectivity between networks, less focalized connectivity, or less-lateralized FC (Negishi et al., 2011; Morgan et al., 2012, 2017). Therefore, in this study, we aim to explore the entire brain network connectivity. Considering that brain regions involved in the FCs will be closely dependent on the etiology and the focal epileptogenic regions, only MTS patients were included in this study.

FC differences can demonstrate alterations in brain networks leading to different patient presentations and may be able to predict patient outcomes after surgery via non-invasive imaging techniques. In this retrospective study, we explore the unique differences in FC patterns of TLE-MTS patients who either (1) experienced FA seizures or FIA seizures pre-operatively, and (2) were classified as either seizure-free or not seizure-free, 12 months postoperatively. All interrogated patients suffered from left-sided TLE-MTS and had proceeded with LiTT as a part of their treatment plan between the years of 2012–2021 at Thomas Jefferson University Hospital (Philadelphia, PA USA).

## Materials and methods

### Subject demographics

FC metrics were ultimately obtained from the comparisons of rsfMRIs amongst our various patient populations. All patients in this cohort were confirmed to have TLE-MTS. The clinical diagnosis of TLE-MTS was made in accordance with criteria from the International League Against Epilepsy (ILAE) (Scheffer et al., 2017). Preoperative assessments consisted of their history, semiology, neuropsychological evaluation, video electroencephalography, as well as advanced neuroimaging modalities including intracranial electroencephalography, 18F-FDG PET, structural and functional MRI (Atsina et al., 2017). The combination of these techniques contributed to their diagnosis of TLE-MTS, localization of the epileptogenic zone, and assessment of candidacy for LiTT.

Below, we describe our two overarching investigations: the first which focused on 12-month postoperative outcomes, and the second, on semiology. Patients were deemed to be SF 12 months postoperatively based upon the Engel Epilepsy Surgery Outcome Scale, where Class I describes the patient as being completely free of disabling seizures. The diagnosis regarding semiology, specifically whether the patient endured FA or FIA seizures, was made on clinical grounds with support from EEG findings. The demographics for both inquiries, in addition to the data regarding the duration of epilepsy for these patient populations, are further detailed in Table 1. All patients in this study were taking antiseizure medications at the 12-month follow-up.

**Table 1:** Subject demographics are categorized by post-operative outcome or semiology, including age and duration of epilepsy.

	**Age (years)**		**Duration of Epilepsy (years)**	
**Population**	**Mean** ±**SD**	**Range**	**Mean** ±**SD**	**Range**
Healthy Participants	24.94 ± 4.28	21–35	–	–
Seizure Free (SF)	47 ± 11.91	31–66	24.87 ± 15.15	4–56
Not Seizure Free (nSF)	48.94 ± 16.94	23–69	24.56 ± 18.99	2–61
Focal Aware Seizures (FA)	47.31 ± 13.76	24–68	21.92 ± 16.77	4–56
Focal Impaired Awareness Seizures (FIA)	47.30 ± 13.51	24–66	27.90 ± 17.16	8–60

**Figure 1:**
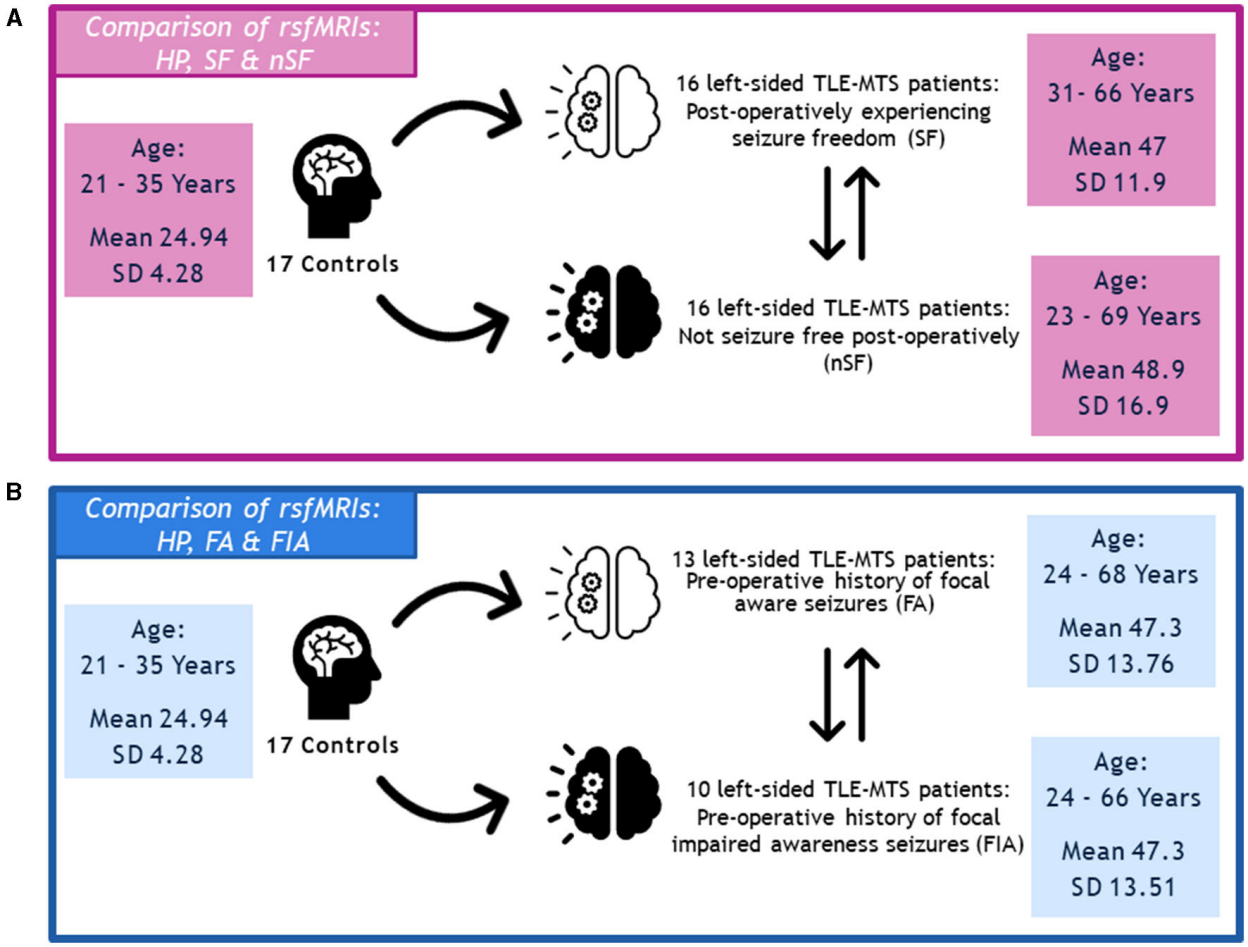
**(A)** Depiction of methodology regarding the comparison of rsfMRIs between the healthy participants, seizure-free (SF) group, and not seizure-free (nSF) group, and **(B)** the comparison of rsfMRIs between the healthy participants, focal aware (FA) group, and focal impaired awareness (FIA) group.

In terms of postoperative outcomes, our left-sided TLE-MTS patients were either categorized as SF (*n* = 16, age 31–66 years, mean age 47 years ± 11.9) or nSF (*n* = 16, age 23–69 years, mean age 48.9 years ± 16.9 years) as determined during their 12-month post-operative follow-up appointment. The rsfMRIs of both groups were compared against those from our cohort of 17 healthy participants (aged 21–35 years, mean age 24.94 years ± 4.28 years), as well as to each other (Figure 1).

Regarding semiology, we specifically explored our left-sided TLE-MTS patient populations with FA seizures (*n* = 13, age 24–68 years, mean age 47.3 years ± 13.76 years) and FIA seizures (*n* = 10, age 24–66 years, mean age 47.3 ± 13.51 years). Similarly, the rsfMRIs of these two groups were compared against the healthy participants (HP) described above, as well as against each other (Figure 1).

### Imaging protocol

Resting-state fMRI data were obtained from all the participants using a 3T Philips Achieva MR scanner with an 8-channel head coil. rsfMRI images were acquired axially using a single-shot echo planar imaging (EPI) sequence to examine the intrinsic functional connectivity of the brain regions. fMRI data were collected from the whole brain prescribed by 3D T1 Magnetization-Prepared Rapid Gradient-Echo (MPRAGE) scans. The T1-MPRAGE imaging parameters used were: FOV = 24.0 cm, voxel size = 1.0 × 1.0 × 1.0 mm^3^, matrix size = 512 × 512, TR = 12 ms, TE = 6 ms, and slice thickness = 1 mm. Resting-state imaging parameters were FOV = 23.0 cm, voxel size = 3.0 × 3.0 × 3.0 mm^3^, matrix size = 128 × 128, TR = 2.5 s, TE = 62 ms, number of averages = 1 and acquisition time = 12 min (360 volumes). Participants were instructed to relax, keep their eyes open and think of nothing in particular during the resting-state scan.

### Data preprocessing

Preprocessing was carried out on the publicly available, MATLAB-based CONN toolbox (Whitfield-Gabrieli and Nieto-Castanon, 2012) in conjunction with SPM12 following the recommended processing pipeline. Structural and functional raw data were visually inspected for quality control. Next, functional images were corrected for temporal differences by slice-timing, and for head motion by realignment and co-registration to the patient's T1-weighted scan. Co-registration and resampling of functional data to a reference image addresses potential distortion artifacts due to head movement. This is accomplished by estimating the derivatives of the deformation field with respect to head movement, to match the deformation field of the reference image. The amount of subject-motion and global BOLD signal were used for outlier removal. Acquisitions with framewise displacement (FD) above 0.9 mm or global BOLD signal changes above the 5 standard deviations (SD) were flagged as outliers and removed from the data. Data were then normalized to the Montreal Neurological Institute (MNI) space, spatially smoothed with an 8-mm full-width half-maximum Gaussian kernel, and denoised based on anatomical component analysis correction (aComCor) to avoid spurious results caused by increased sensitivity of rsfMRI to physiological and extraneous noise. In addition, resting-state time series were linearly detrended, and bandpass filtered (0.008–0.09 Hz) to select for low-frequency components and to reduce low-frequency drift, noise effects, and the confounding influence of respiratory (~0.3 Hz) and cardiac (~1 Hz) noise.

For each of the aforementioned group-based comparisons, a ROI-to-ROI connectivity analysis was pursued via the CONN toolbox. This analysis encompassed a total of 175 regions of interest (ROIs) based upon a customized derivation of the Automated Anatomical Labeling Atlas 3 (AAL3). The AAL atlases are based on a normalized brain in MNI space. Our alterations to the traditional AAL3 atlas included additional regions such as the left and right pendunculopontine nucleus, the left and right pontomedullary reticular formation, and the brainstem. The analysis accounted for covariates such as individual age at MRI acquisition and duration of epileptic activity (Table 1); however, neither covariate was deemed significant in comparisons between the FA and FIA groups, nor between the SF and nSF groups. Bonferroni correction (α = 0.05) was used to correct for multiple comparisons. Significant correlations for both seizure outcomes and semiology were determined via pairwise comparisons.

## Results

Patient sex was found to have no association with semiology nor seizure freedom status 12 months post-operatively, as determined by the Chi-Square Test of Independence. Moreover, neither age of onset nor duration of epilepsy was found to be significant between the SF and nSF groups, or between the FA and FIA groups, as determined by a two-sample *t-*test.

Please note that our patient population specifically included those with left-sided TLE-MTS, and as such, throughout this work, ipsilateral will refer to the patient's left side.

### Global connectivity matrices

**Figure 2:**
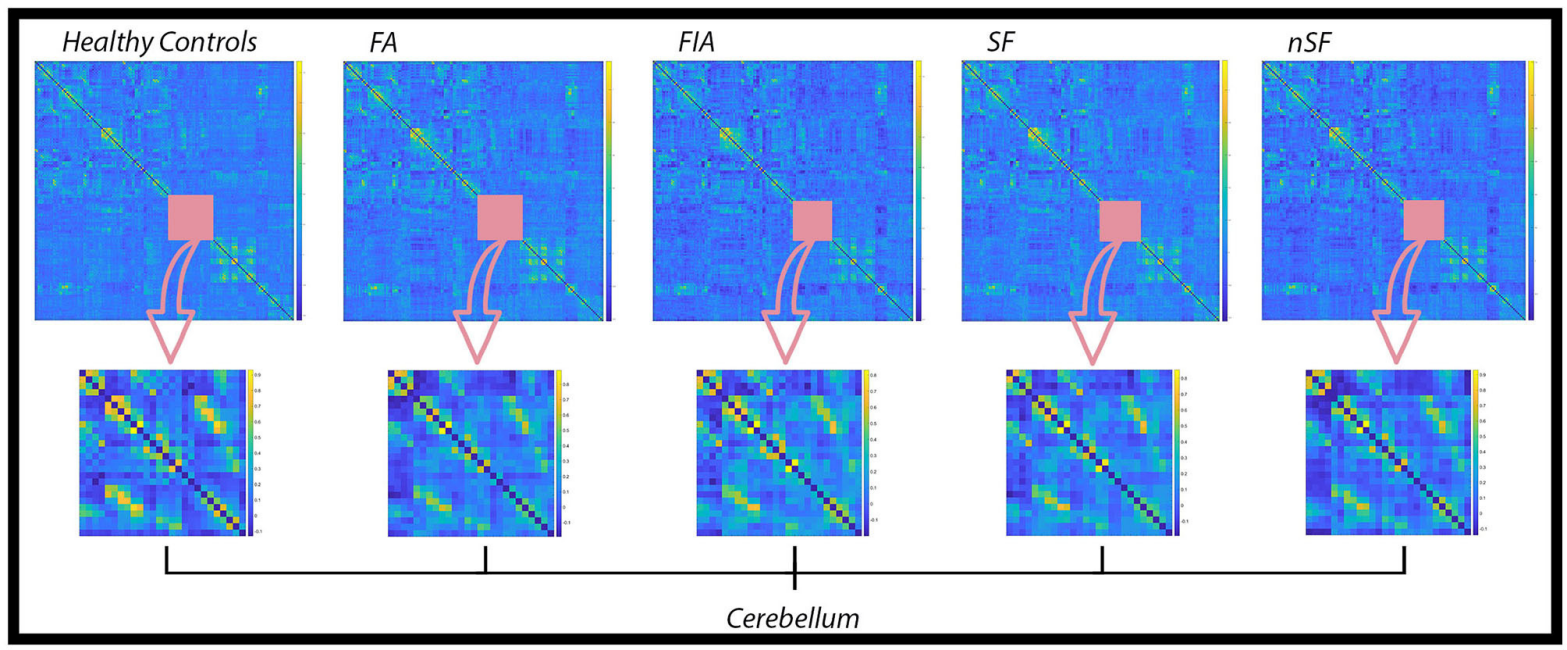
Global Connectivity Matrices (GCM) for the healthy controls, FA, FIA, SF, and nSF populations. In each of these GCMs, regions constituting the cerebellum are highlighted by the pink square and emphasized in the row below. Noticeable variations in the connectivity patterns within the cerebellum across each population denote local alterations in addition to the overall global differences observed.

Global connectivity matrices (GCM) were computed for the cohort of healthy participants, as well as for the FA, FIA, SF, and nSF groups (Figure 2). GCM are useful for the overall screening of averaged connectivity, particularly as they aid in the depiction of group-specific differences. Each entry of the GCMs contains an estimate of the functional connectivity between neural regions listed on the horizontal and vertical axes, which is calculated as the correlation between the time series data of said regions (Venkatesh et al., 2020). Not only were global differences observed across the GCMs for each group, but focusing solely on the cerebellum across all of the GCMs, for example, revealed local alterations as well (Figure 2).

### Post-operative outcomes investigation

**Figure 3:**
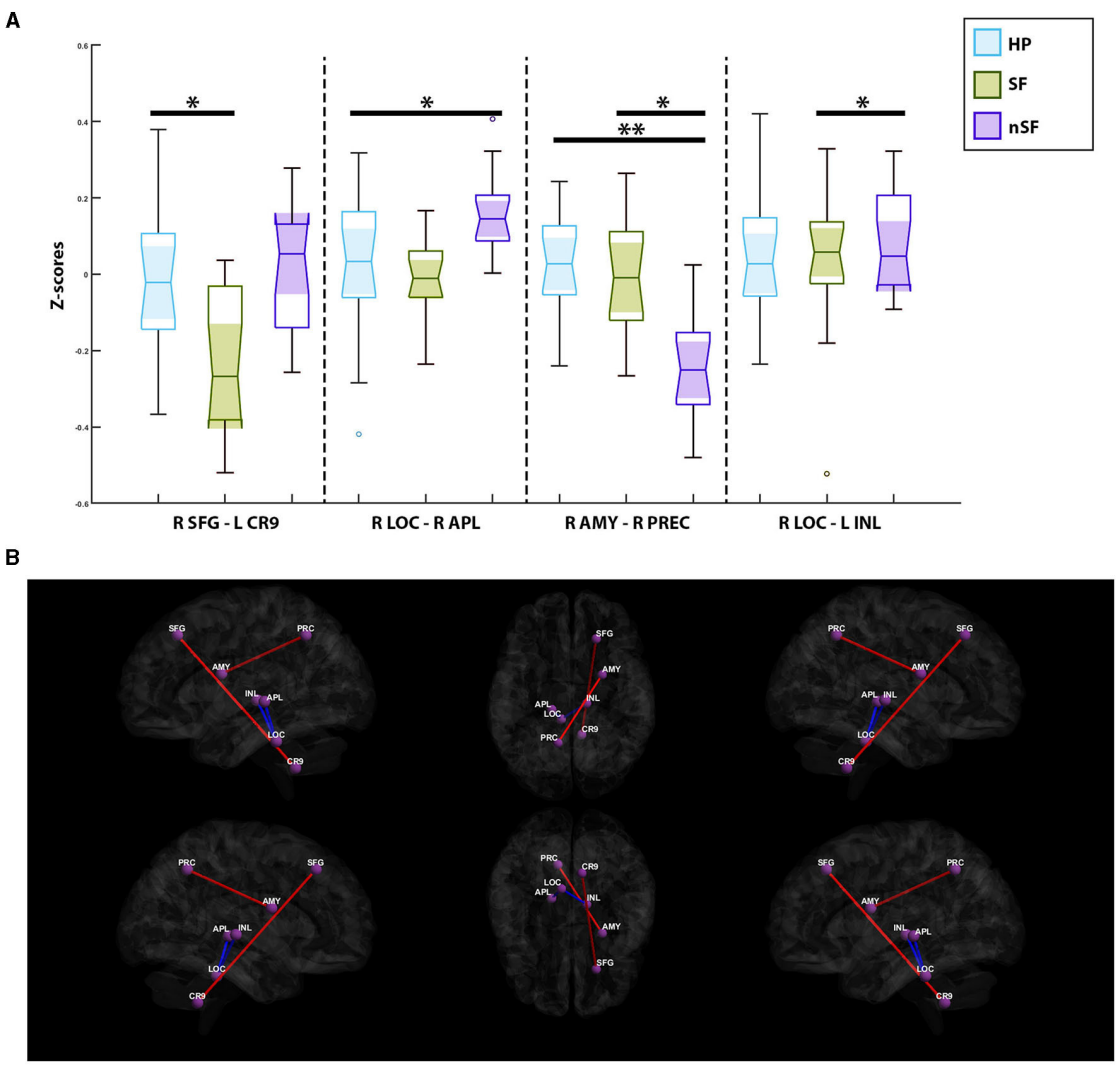
**(A)** Notched box-plots depicting the decreased and increased connectivity between significantly paired-ROIs for each of the postoperative outcome-based comparisons; healthy participants vs. nSF, healthy participants vs. SF, and SF vs. nSF. **(B)** Visualization of functional connectivity across the healthy controls, SF and nSF populations; blue colors indicate a decreased connectivity between paired-ROIs, while the red colors indicate increased connectivity. SFG, Contralateral Superior Frontal Gyrus; CR9, Ipsilateral Cerebellar Hemisphere; Lobule IX, AMY, Contralateral Amygdala; PRC, Contralateral Precuneus; LOC, Contralateral Locus Coreuleus; APL, Contralateral Anterior Pulvinar; INL, Ipsilateral Intralaminar Nucleus. *Symbolizes a significant correlation between two groups of subjects; **Depicts an additional significant correlation between two other groups of subjects.

A single pair of ROIs was deemed significant when comparing the healthy participants and the SF group. Increased connectivity was observed in the healthy participants between the contralateral superior frontal gyrus and the ipsilateral cerebellar hemisphere, Lobule IX (*p*-value = 1.14e-4, mean z-score = −0.11 ± 0.21, T = 4.46). In comparisons between the healthy participants and nSF group, two paired-ROIs were found to be significant: the contralateral amygdala with the contralateral precuneus (*p*-value = 1.1e-5, mean z-score = −0.11 ± 0.19, T = 5.29), and the contralateral locus coeruleus with the contralateral anterior pulvinar (*p*-value = 5.7e-5, mean z-score = 0.09 ± 0.17, T = −4.71). The healthy participants were found to exhibit increased connectivity in the former, and decreased connectivity in the latter (Figure 3).

Comparisons between the SF and nSF groups depicted two significant paired-ROIs; the SF group had shown increased connectivity between the contralateral amygdala and the contralateral precuneus (*p*-value = 2.9e-5, mean z-score = −0.12 ± 0.19, T = 4.98). However, between the contralateral locus coeruleus and the ipsilateral intralaminar nucleus (*p*-value = 1.37e-4, mean z-score = 0.06 ± 0.17, T = −4.41), decreased connectivity in the SF group was observed (Figure 3).

### Semiology investigation

**Figure 4:**
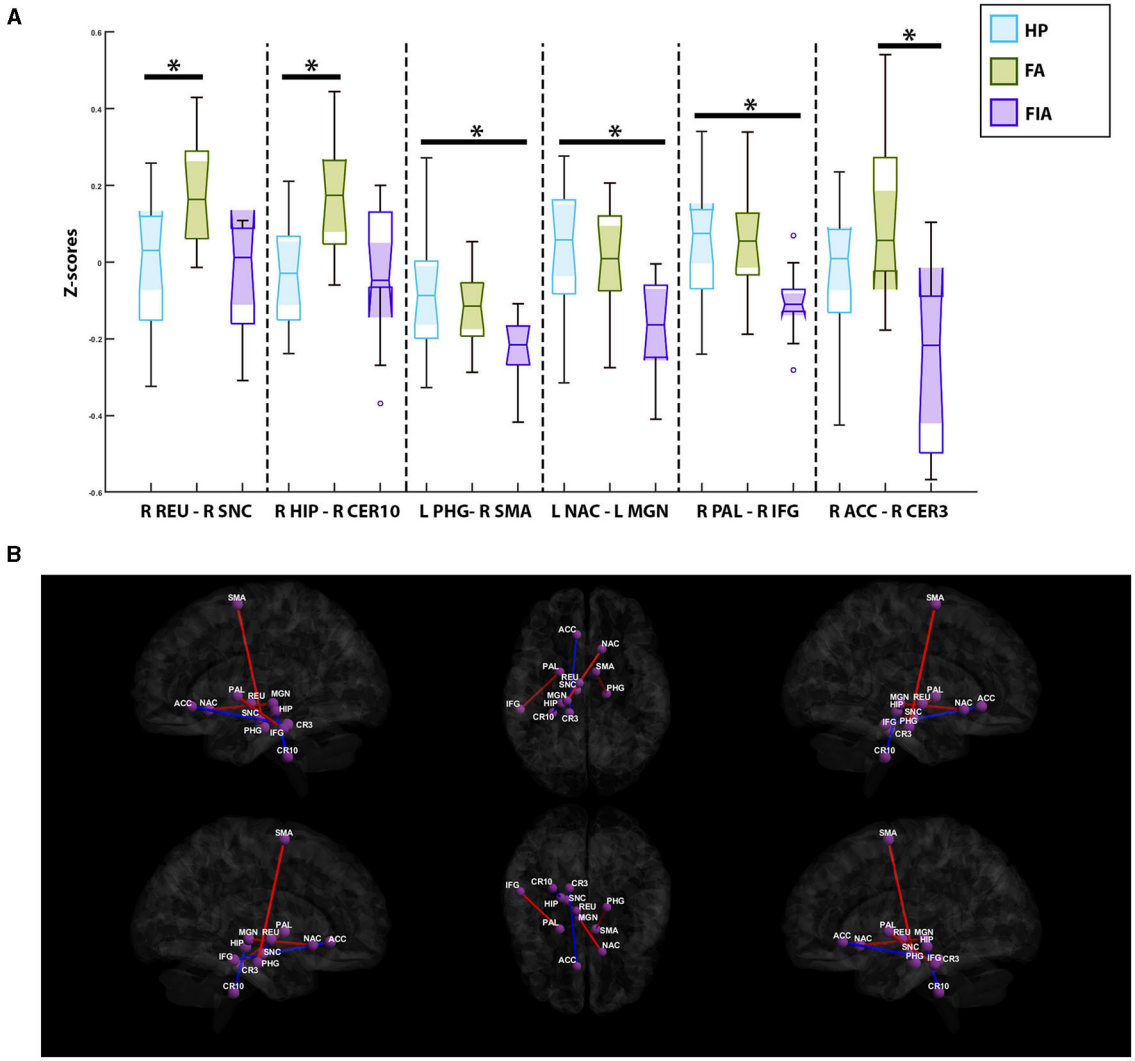
**(A)** Notched box-plots depicting the relative decreased and increased connectivity between significantly paired-ROIs for each of the semiology-based comparisons; healthy participants vs. the focal aware (FA) seizure cohort, healthy participants vs. the focal impaired awareness (FIA) seizure group, and FIA vs. FA. **(B)** Visualization of functional connectivity across the healthy controls, FA, and FIA populations; blue colors indicate a decreased connectivity between paired-ROIs, while red colors indicate increased connectivity. PHG, Ipsilateral Parahippocampal Gyrus; SMA, Contralateral Supplementary Motor Area; NAC, Ipsilateral Nucleus Accumbens; MGN, Ipsilateral Medial Geniculate Nucleus; PAL, Contralateral Pallidum; IFG, Contralateral Inferior Temporal Gyrus; REU, Contralateral Reuniens Nuclueus; SNC, Contralateral Substantia Nigra Pars Compacta; HIP, Contralateral Hippocampus; CER10, Contralateral Cerebellum; Lobule X, ACC, Contralateral Anterior Cingulate Gyrus; Subgenual Region, CER3, Contralateral Cerebellum, Lobule III. *Symbolizes a significant correlation between two groups of subjects.

In comparing the healthy participants with the FIA group, three paired-ROIs were deemed significant: the ipsilateral parahippocampal gyrus and the contralateral supplemental motor area (SMA) (*p*-value = 5.1e-5, mean z-score = −0.14 ± 0.15, T = 4.75), the ipsilateral nucleus accumbens and the ipsilateral medial geniculate nucleus (*p-*value = 1.53e-4, mean z-score = −0.04 ± 0.18, T = 4.35), and the contralateral pallidum with the contralateral inferior temporal gyrus (*p-*value = 1.58e-4, mean z-score = −0.009 ± 0.15, T = 4.34). Across all three of these paired-ROIs, the healthy participants exhibited increased connectivity in comparison to the FIA group (Figure 4). In contrast, only two paired-ROIs were determined to be significant in comparisons between the healthy participants and the FA group. These two paired-ROIs included the contralateral reuniens nuclueus and the contralateral substantia nigra pars compacta (*p*-value = 6.9e-5, mean z-score = 0.08 ± 0.18, T = −4.64), as well as the contralateral hippocampus and the contralateral cerebellum, lobule X (*p*-value = 1.77e-4, mean z-score = 0.05 ± 0.17, T = −4.30). In both pairs, the healthy participants experienced decreased connectivity when compared to the FA group (Figure 4). Lastly, comparisons of the FA group and the FIA group depicted a significant correlation between the contralateral anterior cingulate gyrus, subgenual region, and the contralateral cerebellum, lobule III (*p*-value = 2.26e-4, mean z-score = −0.05 ± 0.28, T = −4.23). Here, it was noted that the FA group experienced increased connectivity in comparison to the FIA group (Figure 4).

## Discussion

In this proof-of-concept study, we sought to examine individuals with TLE-MTS in order to determine functional connectivity alterations in SF and nSF patients, as well as patients experiencing FA and FIA seizures. Both regional and global FC between pairs of atlas-identified ROIs were explored to ensure that entire brain networks were included. At the very least, our results are consistent with other groups in the sense that functional disruption in TLE spreads throughout the cortex beyond the temporal lobe, implying greater diversity in the affected populations (Dumlu et al., 2020).

### Seizure freedom

More specifically, we found that on a global level, SF and nSF patients presented with network disruptions in the cerebellar, limbic, and thalamo-arousal networks. Compared to the healthy controls, FC between the contralateral superior frontal gyrus and ipsilateral lobule IX of the cerebellar hemisphere was significantly decreased in the SF group, whereas it likely increased in the nSF group. Although few studies have assessed the involvement of the cerebellum in epilepsy, BOLD responses in the cerebellar, along with mesolimbic, networks have been reported in TLE-MTS patients (Dansereau et al., 2014). In addition, it was suggested that epileptic discharges from the frontal focus have the tendency to spread toward the cerebellum (Fahoum et al., 2012), while epileptic discharges from the orbital-frontal focus extend toward the mesial temporal structures (Smith et al., 2004). Our findings further suggest a possible involvement of the cerebellum in TLE-MTS, as well as its potential as a biomarker to predict surgical outcomes.

On a regional level, compared to the SF and healthy controls, nSF demonstrated increased connectivity in the thalamo-arousal network as indicated by significantly increased inter-regional FC between the contralateral locus coeruleus of the brainstem and both the contralateral anterior pulvinar nucleus of the thalamus and the ipsilateral intralaminar nucleus of the thalamus. Abnormal thalamic connections leading to hyperactive seizure spread networks were also found by Sathe et al. (2022), similarly contributing to the patients' poor response to epilepsy surgery. In fact, improvements in the thalamo-arousal network connectivity disturbances were observed by González et al. (2019) in those who achieved seizure freedom or reduced seizure frequency following surgery.

Despite different imaging modalities, such as magnetoencephalography, intracranial electroencephalography, and rsfMRI employed in previous studies, it was found that patients with weaker and relatively more focal FC patterns tend to achieve seizure freedom; on the contrary, patients with increased and more diffuse FC patterns, especially distributed to the contralateral hemisphere, are likely to have persistent seizures or higher risk of seizure recurrence (Antony et al., 2013; Englot et al., 2015; He et al., 2017; Neal et al., 2019). Our present study corroborated the spreading of the network to the contralateral hemisphere in nSF patients. Moreover, laterality-based changes may serve as a possible biomarker. For example, bilateral alterations of FC patterns were only seen amongst the SF as compared to healthy participants, whereas unilateral alterations of FCs were only noted in nSF in comparison to their healthy counterparts.

### Semiology

Previously referred to as Simple Partial seizures, those with FA seizures typically remain awake and aware throughout their episode, and often are able to verbally communicate (Kumar and Sharma, 2022). Conversely, those with FIA seizures, formerly described as Complex Partial seizures, are mainly characterized by enduring a loss of, or altered, consciousness, variable degrees of amnesia, and a range of automatisms (Blair, 2012; Vinti et al., 2021). While these two seizure types are primarily differentiated by semiology, there is still much to uncover regarding their underlying pathophysiology; consequently, in this work we attempted to address some of the inscrutability via FC analysis. Similar to the patterns observed in SF and nSF patients, disruptions in the limbic and cerebellar networks were observed in the FA and FIA populations. Our results also brought to light notable alterations in the basal ganglia.

Considering that both groups often experience sensory auras prior to seizure onset, such as complex auditory hallucinations, visual illusions, and/or the deja vu phenomena, disturbances of the limbic system can be expected. Olfactory hallucinations are also relatively specific for TLE and further pinpoint limbic system alterations as a main contributor to these experiences. Additionally, although less common, certain affective behaviors like sudden bouts of misconstrued fear and sadness are also likely to arise from limbic dysfunction.

Perhaps unexpectedly, limbic system disruptions are also implicated in a variety of automatisms afflicting the FIA population. Oral automatisms for example, such as chewing, swallowing, and lip-smacking, are primarily related to amygdala activation (Vinti et al., 2021). However, motor automatisms, such as dystonic posturing and those involving the hands in fumbling, picking, and fidgeting actions (Blair, 2012; Vinti et al., 2021), can instead be correlated with alterations affecting the basal ganglia. Both “leaving behaviors” and preservative automatisms are likely to have roots in basal ganglia disruptions as well. “Leaving behaviors” describe an individual running out of the house or down the street during a seizure, whereas preservative automatisms define a continuation of complex motor acts initiated prior to seizure onset (i.e., opening and closing a door repeatedly) (Blair, 2012). Moreover, since basal ganglia impairment also contributes to complications with controlling speech, less common automatisms including vocalizations, whistling, and ictal speech may also be attributed to this region.

Our results also depicted both unilateral and bilateral changes in the FIA population when comparing this group to the healthy participants and its FA counterparts. This is in contrast to the exclusively unilateral changes in the functional connectivity patterns experienced by the FA group, as compared with both healthy participants and the FIA population. These insights into laterality can, at the very least, be associated with further consequences of basal ganglia dysfunction. For example, some patients may experience unilateral motor phenomena (i.e., ipsilateral contraction of face or mouth and/or head deviation) while others sustain bilateral motor symptoms in their face or axial muscles (Commission on Classification Terminology of the International League Against Epilepsy, 1989). Our analysis revealed that, in contrast to the FIA group and healthy controls, the FA population displayed increased connectivity between all of the significantly correlated paired regions it was implicated in, thus influencing the limbic, cerebellar, and basal ganglia-based networks of those patients to some degree. Perhaps then, it is the comparatively decreased FC signatures that FIA individuals sustain, that lend themselves to the manifestation of the wide variety of automatisms described above. To elucidate this hypothesized relationship further, future work would have to consider each individual's specific symptomatology, and accordingly, analyze the FC patterns of those falling into similar classifications.

Forthcoming iterations of this work would also benefit from larger sample sizes, ideally resulting from multi-center collaborations. The relatively small cohort investigated in this study constrains our investigation to be proof-of-concept in nature, and further restricted our analysis due to the inability of age or sex-matching our subjects. Our population was also not differentiated by whether they endured tonic-clonic episodes in addition to their predominant seizure presentation of FA or FIA, as that would have decreased our sample size significantly. However, taking tonic-clonic episodes into consideration would undoubtedly provide compelling results, given its distinct semiology, and thus should be examined at a later time. Additionally, details regarding educational attainment for this patient population were not collected. In future studies, having this information could further elucidate underlying network connectivity patterns, given its documented influence (Shen et al., 2018). Provided that larger populations could be investigated, a multivariate regression analysis should then be employed to control for the effects of seizure frequency, distribution of seizure types, medication, educational attainment, and other facets. Finally, subsequent investigations seeking to discern potential group differences based on ROI connectivity from the mesial temporal epileptic focus would be of value, particularly since this region is expected to be affected by surgical intervention.

## Conclusion

Our proof of concept FC analysis suggests unique signatures with respect to seizure types as well as 12-month postoperative epileptic outcomes. Our results advise further exploration of the roles the cerebellum, basal ganglia, and limbic system play in TLE-MTS. Moreover, our investigation proposes that global connectivity patterns may have the potential to serve as a “fingerprint” for seizure species; while our work verifies its effectiveness concerning semiology, the technique may also be applied to onset zone delineation as well as potentially determining surgical success. All in all, further explorations of both FC analysis and global connectivity patterns may allow for understanding the differences between epileptic phenotypes, ultimately contributing to targeting advancements.

## Data availability statement

The raw data supporting the conclusions of this article will be made available by the authors, without undue reservation.

## Ethics statement

The studies involving humans were approved by Thomas Jefferson University IRB. The studies were conducted in accordance with the local legislation and institutional requirements. The participants provided their written informed consent to participate in this study.

## Author contributions

MS: investigation, formal analysis, writing—original draft preparation, and writing—review and editing. JM: writing—original draft preparation and data acquisition. ASa and KK: writing—original draft preparation. AM, CM, and ASh: data acquisition. MK: writing—review and editing and data acquisition. MA: conceptualization, methodology, supervision, and writing—review and editing. All authors contributed to the article and approved the submitted version.
