# Supplementary material for: Profiles of resting state functional connectivity in temporal lobe epilepsy associated with post-laser interstitial thermal therapy seizure outcomes and semiologies
Source: Front Neuroimaging. 2023 Nov 9;2:1201682. doi: 10.3389/fnimg.2023.1201682 (PMC10665565; doi:10.3389/fnimg.2023.1201682)
Supplement: Supplementary file 1 [file Data_Sheet_1.PDF]

Table 2. Statistical outputs for the FC signatures of the interrogated populations.

Controls vs. SF		***p-value***	***Z-score (Mean*** ± SD)	***T-value***
Contralateral Superior Frontal Gyrus (SFG)	Ipsilateral Cerebellar Hemisphere, Lobule IX (CR9)	1.14e-4	-0.11 ± 0.21	4.46
Controls vs. nSF				
Contralateral Amygdala (AMY)	Contralateral Precuneus (PREC)	1.15e-5	-0.11 ± 0.19	5.29
Contralateral Locus Coreuleus (LOC)	Contralateral Anterior Pulvinar (APL)	5.7e-5	0.09 ± 0.17	-4.71
SF vs. nSF				
Contralateral Amygdala (AMY)	Contralateral Precuneus (PREC)	2.9e-5	-0.12 ± 0.19	4.98
Contralateral Locus Coreuleus (LOC)	Ipsilateral Intralaminar Nucleus (INL)	1.37e-4	0.06 ± 0.17	-4.41

Controls vs. FIA		***p-value***	***Z-score (Mean ±*** SD)	***T-value***
Ipsilateral Parahippocampal Gyrus (PHG)	Contralateral Supplementary Motor Area (SMA)	5.1e-5	-0.14 ± 0.15	4.75
Ipsilateral Nucleus Accumbens (NAC)	Ipsilateral Medial Geniculate Nucleus (MGN)	1.53e-4	-0.04 ± 0.18	4.35
Contralateral Pallidum (PAL)	Contralateral Inferior Temporal Gyrus (IFG)	1.58e-4	-0.009 ± 0.15	4.34
Controls vs. FA				
Contralateral Reuniens Nuclueus (REU)	Contralateral Substantia Nigra Pars Compacta (SNC)	6.9e-5	0.08 ± 0.18	-4.64
Contralateral Hippocampus (HIP)	Contralateral Cerebellum, Lobule X (CER10)	1.77e-4	0.05 ± 0.17	-4.30
FIA vs. FA				
Contralateral Anterior Cingulate Gyrus, Subgenual Region (ACC)	Contralateral Cerebellum, Lobule III (CER3)	2.26e-4	-0.05 ± 0.28	-4.23

Table 3. Montreal Neurological Institute (MNI) coordinates (X, Y, Z) for significant ROIs as collected from the customized atlas utilized in this work. The customized atlas was derived from the Automated Anatomical Labelling Atlas 3 (AAL3).

Controls vs. SF			
Contralateral Superior Frontal Gyrus (SFG)	(22, 31, 44)	Ipsilateral Cerebellar Hemisphere, Lobule IX (CR9)	(10, −49, −46)
Controls vs. nSF			
Contralateral Amygdala (AMY)	(−27, 1, −18)	Contralateral Precuneus (PREC)	(−10, −56, 44)
Contralateral Locus Coreuleus (LOC)	(−7, −36, −28)	Contralateral Anterior Pulvinar (APL)	(−15, −28, −1)
SF vs. nSF			
Contralateral Amygdala (AMY)	(−27, 1, −18)	Contralateral Precuneus (PREC)	(−10, −56, 44)
Contralateral Locus Coreuleus (LOC)	(−7, −36, −28)	Ipsilateral Intralaminar Nucleus (INL)	(14, −23, 0)

Controls vs. FIA			
Ipsilateral Parahippocampal Gyrus (PHG)	(18, −18, −21)	Contralateral Supplementary Motor Area (SMA)	(9, 0, 62)
Ipsilateral Nucleus Accumbens (NAC)	(14, 19, -9)	Ipsilateral Medial Geniculate Nucleus (MGN)	(−15, −24, −5)
Contralateral Pallidum (PAL)	(−21, 0, 0)	Contralateral Inferior Temporal Gyrus (IFG)	(−54, −31, −22)
Controls vs. FA			
Contralateral Reuniens Nuclueus (REU)	(−5, −9, −5)	Contralateral Substantia Nigra Pars Compacta (SNC)	(−7, −15, −15)
Contralateral Hippocampus (HIP)	(−20, −26, −10)	Contralateral Cerebellum, Lobule X (CER10)	(−27, −34, −41)
FIA vs. FA			
Contralateral Anterior Cingulate Gyrus, Subgenual Region (ACC)	(−7, 31, −7)	Contralateral Cerebellum, Lobule III (CER3)	(−13, −34, −19)
